# The Effect of the Boar Taint Masking Strategy (Adding Dried *Origanum vulgare* or *Allium sativum)* on Sensory Characteristics

**DOI:** 10.3390/ani14111544

**Published:** 2024-05-23

**Authors:** Kateřina Zadinová, Adam Sochor, Jaroslav Čítek, Monika Okrouhlá, Kamila Pokorná, Michal Šprysl, Ivan Bahelka, Roman Stupka

**Affiliations:** Department of Animal Science, Faculty of Agrobiology, Food and Natural Resources, Czech University of Life Sciences Prague, Kamýcká 129, 165 00 Prague, Czech Republic; adamsochor@seznam.cz (A.S.); citek@af.czu.cz (J.Č.); okrouhla@af.czu.cz (M.O.); pokornakamila@af.czu.cz (K.P.); sprysl@af.czu.cz (M.Š.); bahelka@af.czu.cz (I.B.); stupka@af.czu.cz (R.S.)

**Keywords:** sensory characteristics, skatole, pork, *Allium sativum*, *Origanum vulgare*

## Abstract

**Simple Summary:**

The aim of this study was to test the possibility of masking boar taint or skatole levels by adding dried *Origanum vulgare* or *Allium sativum* in two experiments and to evaluate the sensory preferences towards the skatole concentration in different carcass parts (*longissimus lumborum*; *semimembranosus*; neck chop and subcutaneous fat). The results of the first experiment showed that the percentages of samples with low (0.069 µg/g) and medium (0.269 µg/g) concentrations of skatole differed significantly between the control group and the groups treated with *Origanum vulgare* or *Allium sativum*. According to the results of the second experiment, meat samples from leaner parts, such as the *semimembranosus*, *longissimus lumborum*, and neck chop not treated with *Allium sativum* and *Origanum vulgare* for masking, were significantly worse in terms of the occurrence of boar taint or abnormal odour. No significant differences were found between the two masking methods.

**Abstract:**

With increasing efforts to ban surgical castration, it is important to find ways to mask the level of boar taint in meat. The aim of this study was to test the possibility of masking boar taint or skatole levels by adding dried *Origanum vulgare* or *Allium sativum* and to evaluate consumer sensory preferences towards the skatole concentration in different carcass parts (*longissimus lumborum*; *semimembranosus*; neck chop and subcutaneous fat) and the masking strategy (addition of *Allium sativum* or *Origanum vulgare*). In the first experiment, the effect of the masking strategy was evaluated at three different skatole concentrations (0.069, 0.269 and 0.463 µg/g). The results showed that the samples with low and medium skatole levels were significantly different between the control group and the groups treated with *Origanum vulgare* or *Allium sativum*. In both cases, the addition of *Allium sativum* and *Origanum vulgare* had a positive effect on the parameters of abnormal odour and pleasantness of odour (*p* < 0.05). According to the results of the second experiment, meat samples from leaner parts, such as the neck chop *semimembranosus* and *longissimus lumborum*, not treated with *Allium sativum* and *Origanum vulgare* for masking, were significantly (*p* < 0.05) worse in terms of the occurrence of boar taint or abnormal odour than the masked samples. No significant differences were found between the two masking methods.

## 1. Introduction

Surgical castration of boars may be banned in many European countries, leading to the problem of boar taint in pork [1,2,3]. Boar taint is an unpleasant odour in pork caused by three main compounds: androstenone (5a-androst-16-ene-3-on), skatole (3-methylindole) and indole [4,5,6,7,8,9]. Androstenone is a steroid hormone produced by Leydig cells in the testis. Skatole and indole are produced by tryptophan metabolism in the large intestine, and these compounds are stored in fat tissue [7,9]. Boar taint is released after heating this tissue and is described as having a sweaty, urine or faecal-like odour [7,10]. Boar taint is considered unacceptable for consumers in many cases [11]. Consumer perceptions of acceptable levels of boar taint compounds have been shown to differ. Skatole is acceptable at concentrations ranging from 0.15 to 0.25 μg/g, and androstenone is acceptable at concentrations ranging from 0.50 to 1.00 μg/g [12,13]. Consumers are a very important part of the production chain; thus, it is necessary to find a way to minimize the perception of boar taint in pork and pork products. The perception of boar taint compounds widely depends on the method of meat preparation [14,15]. The presence of boar taint gives rise to the possibility of boar taint masking. Different types of boar taint masking strategies appear to effectively reduce boar taint perception in both pork and pork products. Masking strategies include the use of different types of herbs and spices, such as garlic (*Allium sativum*), bay leaves (*Laurus nobilis*), clove (*Syzygium aromaticum*), oregano (*Origanum vulgare*), nutmeg (*Myristica fragans*), marjoram herb (*Origanum majorana*), coriander (*Coriandrum sativum* L.) and ginger (*Zingiber officinale*) [16,17,18]. In addition to spices, smoke has often been recommended as a good masking agent for boar taint. However, smoke is primarily applied to pork products, such as bacon, salami and sausages, and not for pork itself [19,20,21]. Martínez et al. [20] recommend the use of a combination of smoke, spices and herbs, such as mustard seed (*Sinapsis alba*), paprika (*Capsicum annum*), nutmeg (*Myristica fragans*), coriander seed (*Coriandrum sativum* L.) and marjoram herb (*Origanum majorana*). Especially garlic, but currently also oregano, is a spice that is often used in Central and Eastern European cuisine. However, most studies have evaluated masking strategies in pork products, not in pork. In Central Europe, individual carcass parts are often used for the preparation of traditional culinary dishes. The selection of appropriate spices typical of the region can allow the use of pork even from uncastrated boars. Similar to culinary preparation, carcass parts can influence the perception of boar taint odour. It has previously been shown that lots with a higher fat content can exhibit poorer sensory characteristics than those with a low fat content [3]. However, unpleasant odours have also been described in relatively low-fat tissues, such as *psoas major* [22]. Meinert et al. [23] evaluated six carcass parts—neck fat, neck (*m. longissimus thoracis*), loin (*m. longissimus thoracis et lumborum*), topside (*m. semimembranosus*), tenderloin (*psoas major*) and eye of round (*m. semitendinosus*)—and showed that parts with a higher fat content are significantly less accepted by consumers because of the presence of boar taint. The reliable sorting and optimized use of boar-tainted pig carcasses are dependent on knowledge of the corresponding sensory characteristics.

Thus, the first aim of this study was to evaluate consumer sensory preferences related to different types of boar taint masking strategies (adding dried *Origanum vulgare* or *Allium sativum)* in pork with different skatole levels. The second aim was to evaluate sensory preferences for different types of masking strategies related to different parts of the carcass (with different fat content—*longissimus lumborum*, *semimembranosus*, muscles from the neck chop and subcutaneous fat).

## 2. Materials and Methods

### 2.1. Animals and Meat Samples Collection

Forty-eight Landrace uncastrated male pigs in total from 12 litters were maintained in a testing station at the Czech University of Life Sciences Prague. All pigs housed on slatted floors in groups of 12 and were fed according to their standard nutrient requirements. The animals were slaughtered at 171 days of age at a commercial slaughterhouse following standard operational procedures and had an average weight of 125.3 ± 4.3 kg. Back fat samples from all boars were collected for the determination of androstenone and skatole levels. The samples of individual carcass parts were collected for sensory analysis and stored as described below. Twelve Landrace boars were selected for sensory analysis based on the levels of androstenone and skatole in the back fat.

The carcasses were cooled at 4 °C for 24 h. Based on the detected values of androstenone and skatole, the samples of individual carcass parts were divided into groups for sensory evaluation. The average androstenone concentration in all the selected samples was 6.577 ± 0.272 µg/g back fat. Skatole and androstenone levels in subcutaneous fat were determined using HPLC/FLD. Samples for skatole and androstenone analyses were collected from pig subcutaneous fat from the neck section between the 1st and 3rd cervical vertebrae one day after slaughter. The samples were vacuum-packed and frozen at –80 °C until analysis at the laboratories of the Department of Animal Science (Czech University of Life Sciences Prague). Meat and subcutaneous fat samples for sensory analysis were sliced at 2 cm, and each sample was individually vacuum-packed and frozen at −20 °C until analysis.

### 2.2. Determination of Skatole and Androstenone Concentrations

Skatole and androstenone levels in the subcutaneous fat were determined by HPLC/FLD (LC-2000Plus HPLC system; Jasco, Tokyo, Japan) using the method described in [24]. The skatole concentration was quantified using a Kinetex C18 100A column (5 µm, 50 × 4.6 mm) (Phenomenex, Torrance, CA, USA) at 40 °C. The mobile phases were potassium phosphate (10 mM; A) and methanol (B). The gradient profile program was 0–0.2 min at 90% phase A, 0.2–6 min at 90–55% A and 6–7 min at 55–0% A. The flow rate was 1.2 mL/minute, and an injection volume of 30 µL was used. Florescence detection was performed at an excitation wavelength of 285 nm and an emission wavelength of 340 nm, and standard curves were produced to quantify the concentrations of skatole and indole. The androstenone concentration was determined using an Agilent Eclipse XDB C18 column (5 µm, 150 × 4.60 mm) (Agilent, Santa Clara, CA, USA) at 40 °C. The two mobile phases used were tetrahydrofuran:acetonitrile:potassium phosphate (25 mM):acetic acid (phase A) and methanol (phase B). The gradient program used was as follows: 0–3 min, 90% phase A; 3.5–15 min, 45–55% phase A; 15–16.1 min, 5% phase A; 16.1–17 min, 5–90% phase A; and 17–19 min, 90% phase A. The flow of the mobile phase was 1.2 mL/minute with a 40 µL injection volume.

### 2.3. Meat Samples for Sensory Analysis

Two hundred and sixteen samples from *longissimus lumborum* were selected for sensory evaluation in the first experiment. The samples were divided into three groups based on average skatole levels in the back fat: low level of skatole, 0.069 ± 0.0016 µg/g (n = 72); medium level of skatole, 0.269 ± 0.0241 µg/g (n = 72); and high level of skatole, 0.463 ± 0.0370 µg/g (n = 72). The skatole concentration for the low group was chosen based on a publication by Stupka et al. [25], who described this level of similar concentration in barrows, gilts and immunocastrates. The group with a medium level of skatole was chosen according to a threshold value acceptable to the consumer according to Bonneau et al. [12] and Aluwé et al. [12], and twice the threshold value was chosen as the high skatole concentration. A separate session was held for each group (skatole level), and four sets of three samples (control, *Allium sativum* and *Origanum vulgare)* were presented to each panellist in each session.

For the second follow-up experiment, 288 samples were collected from four carcass parts: *longissimus lumborum* (n = 72), *semimembranosus* (n = 72), muscles from the neck chop (n = 72) and subcutaneous fat (n = 72) from the dorsal part of the neck. All samples for this analysis were selected based on the average levels of androstenone and skatole. The average level of skatole in all samples was 0.269 ± 0.0241 µg/g (samples from animals in the second group from the first experiment). A skatole level of 0.25 μg/g is considered detectable by most consumers. For this reason, samples with a skatole level of 0.269 ± 0.0241 µg/g were subsequently selected for the second experiment. A separate session was held for each carcass part, and four sets of three samples (control, *Allium sativum* and *Origanum vulgare)* were presented to each panellist in each session.

### 2.4. Selection of Sensory Panellists

All samples were subjected to sensory analysis by a trained sensory panel. Six panellists (3 females and 3 males) were chosen based on their ability to assess androstenone and skatole levels, as described by Meier-Dinkel [26]. The panellists were selected during screening sessions in the sensory laboratory of the Czech University of Life Sciences Prague. The selection process focused on drafting persons who were able to detect androstenone and skatole. Olfactory acuity was determined using triangle tests with paper smell strips. Only panellists who correctly discriminated between very low amounts of androstenone (~10 ng; 20 μL of 0.5 μg/g androstenone dissolved in propylene glycol) and skatole (~20 ng; 20 μL of 1 μg/g dissolved in propylene glycol from the pure solvent) in triplicate triangle tests were selected. Subsequently, a descriptive sensory analysis (DSA) of the meat and fat samples was performed in individual booths under controlled environmental conditions and red light (ISO 8589, 2007).

### 2.5. Preparation of Samples and Descriptive Sensory Analysis (DSA)

The frozen meat and fat samples were thawed for 24 h at 4 °C before sensory analysis. The samples were coated with weighed quantities of dried *Origanum vulgare* and freshly peeled and finely chopped *Allium sativum* immediately before cooking. Four sets of three samples (control, sample with 2.5 g/1000 g dry *Origanum vulgare* and sample with 30 g/1000 g cut *Allium sativum*) were presented to each panellist in each session in both experiments. *Allium sativum* and *Origanum vulgare* are herbs that are often used in Central and Eastern European cuisine.

Subsequently, the samples were vacuum-packaged (Vac-star, 180 × 200 mm, 90 µm thick), labelled and cooked in a water bath at 80 °C for 60 min. Immediately after cooking, the samples were cut into 2 × 2 × 2 cm cubes, excluding the outer meat or fat surface, placed into glass containers and sealed. Each container/sample was marked with a randomized 3-digit code and placed into an oven at 50 °C for 1 h until evaluation. The panellists were provided with water and bread to cleanse their palates between samples. A linear unstructured continuous 100 mm scale oriented by descriptions at both ends was used for each of the seven descriptors defined in Table 1. This scale was then transformed into a numerical scale (0 = cannot be detected−100 = very intense) for statistical analysis.

**Figure 1:**
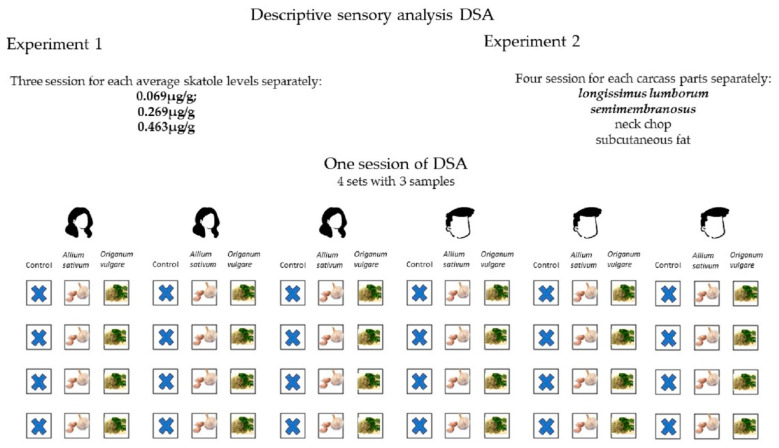
Descriptive sensory analysis scheme.

DSA for the first study was performed over a total of three sessions (for each skatole level) with four sets of three samples of *longissimus lumborum*. DSA for the second study was performed over a total of four sessions (for each carcass part—*longissimus lumborum; semimembranosus*; neck chop and subcutaneous fat) with four sets of three samples (see Figure 1).

Three sessions were conducted in the first experiment. Samples with an average skatole level of 0.069 µg/g were evaluated in the first session, samples with an average skatole level of 0.269 µg/g were evaluated in the second session, and samples with an average skatole level of 0.463 µg/g were evaluated in the third session. Within each session, each panellist was presented with 4 sets of 3 samples of *longissimus lumborum* muscle (control, *Allium sativum* and *Origanum vulgare*). In the second experiment, the effect of the masking strategy on individual carcass parts (neck chop, *longissimus lumborum*, *semimembranosus* and subcutaneous fat) was evaluated. All samples evaluated in this experiment had an average skatole level of 0.269 µg/g. A separate session was conducted for each carcass part. Within each session, 4 sets of 3 samples (control, *Allium sativum* and *Origanum vulgare*) were again presented to each panellist. In the first and second experiments in each session, a total of 24 samples were evaluated for each single masking strategy—four samples for each of the six panellists.

### 2.6. Statistical Analyses

The data provided by the panellists for the descriptive sensory analysis were analysed using the general linear model (GLM) method of the MIXED procedure of the SAS statistical package, version 9.4 (Stat 2015, SAS Institute Inc., Cary, NC, USA). The Shapiro–Wilk test was performed on the standardized residuals from the model to ensure normality, and no outlier values were identified or removed. The model included the fixed effects of carcass parts (neck chop, *longissimus lumborum*, *semimembranosus*, subcutaneous fat), skatole level, masking strategy and the random effects of the assessor. The data in the table are presented as least squares means (LSM) and standard errors of the mean (SEM). Differences were considered significant at the *p* < 0.05 level.

## 3. Results

### 3.1. Evaluating the Effect of Different Types of Boar Taint Masking Strategies on Pork with Different Levels of Skatole

The results of the comparison of the two boar taint masking strategies for *longissimus lumborum* samples as a function of the skatole level are described in Table 2, Table 3 and Table 4.

Differences were observed between the control and *Allium sativum-* or *Origanum vulgare*-treated samples for the groups of samples with the lowest (0.069 µg/g) and medium (0.269 µg/g) skatole levels. For the group of samples with the lowest skatole level (Table 2), there were significant differences (*p* < 0.05) between the control group and the masked samples regarding the following parameters: typical pork odour, pleasantness of odour and typical pork flavour. A significant difference was observed for the parameters of abnormal odour and abnormal flavour (*p* < 0.05) between the control and *Origanum vulgare* samples. In all evaluated parameters, the control group was the worst.

The results recorded in Table 3 for samples with medium skatole levels show the same trend as those with low skatole levels. For all the parameters evaluated, the samples from the control group performed the worst. For this skatole level, masking by *Allium sativum* was slightly better than masking by *Origanum vulgare*, especially for the parameters of abnormal odour (*p* < 0.05) and pleasantness odour (*p* < 0.05); however, the differences in each parameter among the panellists were not significant. No significant differences were observed for the meat flavour or overall acceptability of the sample parameters.

For samples with low and medium levels of skatole, it seems possible to mask boar taint with *Allium sativum* and *Origanum vulgare*. However, for the highest skatole level evaluated (0.463 µg/g), the chosen masking strategies did not achieve significant results (see Table 4). Compared with the control samples, the *longissimus lumborum* samples treated with *Allium sativum* and *Origanum vulgare* better evaluated the pleasantness of the odour parameters. In terms of abnormal parameters, compared with the control group, the *Origanum vulgare* treatment group showed the most significant differences (*p* < 0.05) in abnormal flavours at the two lower skatole levels. At the highest skatole level, a significant difference was observed only between the *Allium sativum treatment* group and the control group. Overall, the samples treated with *Origanum vulgare* were considered by the panellists to be the most acceptable.

### 3.2. Evaluating the Effect of Different Types of Boar Taint Masking Strategies on Different Types of Carcass Parts

Based on the results of the first part of the experiment, which demonstrated that the perception of boar taint could be influenced by the addition of *Allium sativum* or *Origanum vulgare* even in the sample with an average skatole level (0.269 ± 0.241 µg/g), samples from animals with this average skatole level were included in the second part of the experiment. Table 5, Table 6, Table 7 and Table 8 show the effect of the addition of *Origanum vulgare* and *Allium sativum* on individual carcass parts and adipose tissue. For the evaluated muscle samples (neck chop, *semimembranosus* and *longissimus lumborum*), significant differences were detected for some parameters between the control samples and the samples treated with both types of masking. For subcutaneous fat samples, a significant difference was observed only for typical pork flavour between the control group and the group with the addition of *Allium sativum* or *Origanum vulgare.*

As shown in Table 5, the neck chop samples showed significant differences (*p* < 0.05) between the control samples and those with *Origanum vulgare* in terms of the parameters of typical pork odour, pleasantness of odour and typical pork flavour with *Allium sativum*. There were no significant differences between the two types of masking.

Similar results are described for the other two carcass parts evaluated. Additionally, for the *semimembranosus* and *longissimus lumborum*, the differences between the control group and the two masking groups are described. In the case of the *semimembranosus* muscle (see Table 6), significant differences between the control and both masking strategies were described for typical pork odour, abnormal odour, pleasantness of odour and typical pork flavour.

For the *longissimus lumborum* muscle, the situation is similar to that of the *semimembranosus* (see Table 7). Significant differences (*p* < 0.05) were observed between the control group and both additive groups for typical, abnormal and pleasant odours as well as typical pork flavours. Moreover, *Origanum vulgare* also significantly reduced abnormal flavours.

In the case of subcutaneous fat (Table 8), there was a trend similar to that of the other carcasses, but there were no clear differences. Only for the parameter of typical pork flavour did the panellists observe significant differences in the typical pork flavour between the control group and both the *Allium sativum* and *Origanum vulgare* groups.

**Figure 2:**
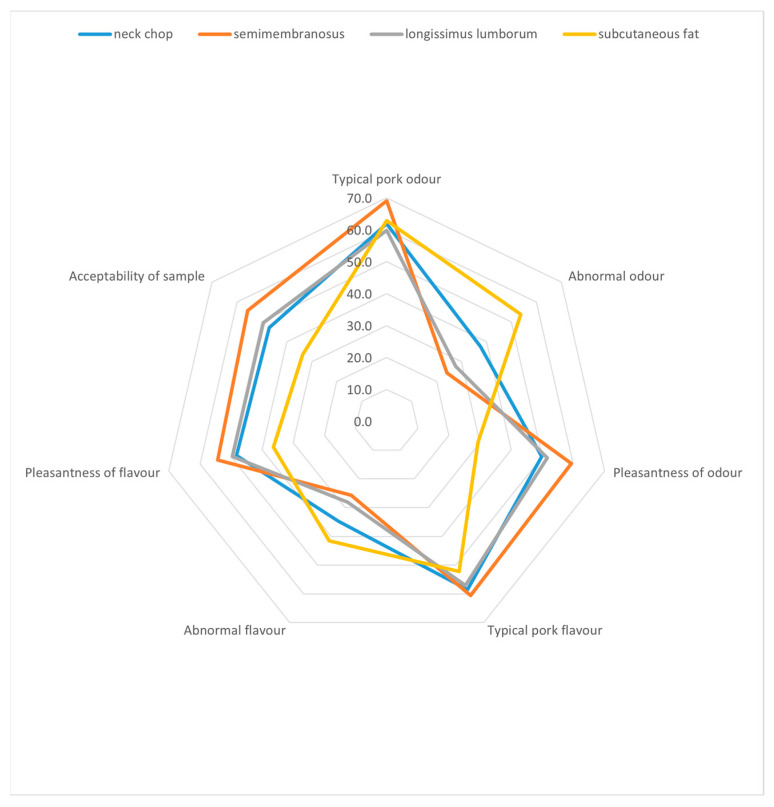
Sensory perception of boar taint in carcass parts.

When comparing the sensory preferences for each carcass part, regardless of the method of camouflage, *semimembranosus* was rated the best by the panellists, and subcutaneous fat was rated the worst, as shown in Figure 2.

## 4. Discussion

### 4.1. Evaluating the Effect of Different Types of Boar Taint Masking Strategies on Pork with Different Levels of Skatole

The selection of boars with the same androstenone concentration was performed to minimize the influence of androstenone on the resulting sensory effect. Bonneau et al. [12] and Aluwé et al. [13] demonstrated that the unacceptable concentration of skatole for consumers is 0.25 μg/g. However, even at lower skatole values, more sensitive individuals can detect deterioration in sensory parameters, although to a limited extent. As Vold [27] and De Kock et al. [28] described, skatole is the cause of not only unpleasant odours but also unpleasant tastes. This is also confirmed in the results of our experiments, where increasing levels of skatole also increased the sensitivity of consumers to abnormal meat flavours. The results of the first experiment showed that the sensory parameters of samples with skatole concentrations of approximately 0.25 μg/g or less can be positively influenced by the addition of *Allium sativum* or *Origanum vulgare*.

The effect of *Origanum* vulgare and *Allium sativum* masking on the given sensory parameters was significant. In the case of abnormal odour and flavour, there was a strong difference between the masked and control samples. The intensity of the abnormal odour was greater in the control samples than in the masked samples, even at the lowest skatole concentration. Thus, it can be concluded that the odour of skatole can be partially eliminated by other aromatic natural compounds, such as allicin from *Allium sativum*. This view was also supported by Lunde et al. [19], who stated that the addition of *Allium sativum* perfectly masks boar taint and that it was impossible to distinguish between the meat or fat of castrated and uncastrated individuals. In contrast to the intensity of the abnormal odour, the pleasantness of the odour increased considerably when *Origanum vulgare* or *Allium sativum* was applied in combination. Thus, the odours of *Origanum vulgare* and *Allium sativum* are more pleasant to most of the population than the odour of skatole.

The positive effect of *Origanum vulgare* or *Allium sativum* addition has also been described in other studies [19,22,26,29], not only for fresh meat but also for meat products, such as sausages and chorizo. However, contrary to our results, previous studies have indicated that *Allium sativum* has a good effect on taste. An increased incidence of abnormal taste was observed in this study. However, it is unclear whether this is due to the low masking effects of *Allium sativum* or the sensitivity of the panellists to *Allium sativum*. The reason could also be that most studies have not used *Allium sativum* alone but rather used a combination of *Allium sativum* and breadcrumbs [19]. Several more recent studies [30,31] have used spice mixtures to mask boar scents. However, better results were achieved with mixtures containing *Allium sativum* than with mixtures in which *Allium sativum* was not included, regardless of how the mixture was applied [30].

### 4.2. Evaluating the Effect of Different Types of Boar Taint Masking Strategies on Different Types of Carcass Parts

Sorting and optimizing the use of boar-tainted pig carcasses depend on the knowledge of the distribution of skatole and the corresponding sensory characteristics. According to a previous study [23], there is a difference between skatole levels in adipose tissue and muscle. However, significant correlations were established between these levels. No significant differences in skatole levels or in the sensory perception of the panellists were detected between the individual muscles. According to more recent studies [9,32], the level of boar taint components in fat, lean tissue and plasma may be affected by the presence of other compounds related to boar taint. One of these compounds is androstenone sulphate, which contributes significantly to the free androstenone content in tissues. Due to the metabolic processes described in these publications, different results in the perception of the different lots can be expected.

In our second experiment, carcass parts such as *longissimus lumborum* and *semimembranosus* were generally acceptable to consumers. This can be explained by the fact that skatole is a lipophilic compound that accumulates predominantly in adipose tissue; therefore, areas with higher fat content can be expected to be more prone to boar taint [33]. The different fat contents in individual parts of the carcass and the increased occurrence of sensory active substances in fat have also been confirmed in previous studies [34,35]. A strong correlation between skatole levels and the fat content of meat cuts was also confirmed by Meinert et al. [23]. A strong correlation was found between the skatole content of the neck fat and the meat cuts evaluated (neck chop—*longissimus thoracis*, *longissimus lumborum*, *semimembranosus*, *psoas major* and *semitendinosus*), although the skatole concentration in the meat cuts was much lower than that in the neck fat. Additionally, different fatty acid compositions in adipose tissue (intramuscular fat, back fat) may influence different perceptions of boar taint or skatole [36]. In our experiment, the masking effect of *Origanum vulgare* or *Allium sativum* reduced the negative perception of boar taint and flavour; however, the parts with higher fat content—the neck chop and subcutaneous fat—were perceived to be worse than *longissimus lumborum* and *semimembranosus*. There were significant differences in the perception of abnormal flavours in *longissimus lumborum* and abnormal odours in *semimembranosus*. Moreover, in terms of the perception of a typical pork odour, a typical pork flavour and flavour pleasantness significantly differed between the control group and the masking strategy for three carcass parts (*longissimus lumborum, semimembranosus* and neck chop). Based on these findings, we can assume that in the case of fattening uncastrated boars, it will be necessary to consider not only the level of skatole but also the fat content. Individual carcass parts can then be used differently.

## 5. Conclusions

Due to the current trend of preventing surgical castration in pigs in Europe, we need to develop ways to reduce boar taint and effectively mask it in pork meat. In conclusion, it is possible to use specific spices, such as *Allium sativum* or *Origanum vulgare*, to mask boar taint or skatole in pork. However, the use of this masking method is only possible at a certain level of skatole. As seen from the results of this experiment, it is possible to mask *Allium sativum* or *Origanum vulgare* by adding them to samples up to an average concentration of 0.269 µg/g. At higher skatole levels, masking with these spices appears to be ineffective. Since skatole, as a lipophilic compound, is mainly deposited in adipose tissue, a lower possibility of masking boar taint or skatole can be expected in carcasses with a higher fat content. Most of the studies published thus far have addressed the masking of boar taint in meat products using highly aromatic spice mixtures. However, the results of the second experiment suggest that at least some carcass parts can be used as meat for culinary preparation using spices typical of the Central European region.

## Figures and Tables

**Table 1:** Definition and scale of attributes used in the sensory analysis to evaluate the meat from different carcass parts and fat from uncastrated male pigs using different masking strategies.

Attribute	Evaluation	Definition
Typical pork odour	Before eating sample	The strength of aroma typical for cooked pork
Abnormal odour	Before eating sample	Intensity of abnormal odours (boar taint or skatole odour)
Pleasantness of odour	Before eating sample	Pleasant or unpleasant
Typical pork flavour	After tasting sample	The strength of flavour typical for cooked pork
Abnormal flavour	After tasting sample	Intensity of abnormal flavours
Pleasantness of flavour	After tasting sample	Pleasant or unpleasant—the panellist subjectively evaluates whether the sample is pleasing (odour/flavour) to him or her or not.
Acceptability of sample	After tasting sample	Panellist’s willingness to consume the sample

**Table 2:** Evaluation of sensory preferences of *longissimus lumborum* in relation to different masking strategies at the skatole level of 0.069 μg/g.

Attribute	Control (n = 24)	*Allium sativum* (n = 24)	*Origanum vulgare* (n = 24)		
	LSM ^1^	LSM ^1^	LSM ^1^	SEM ^2^	*p*-Value
Typical pork odour	50.38 ^b^	68.33 ^a^	76.13 ^a^	3.3	0.026
Abnormal odour	36.58 ^a^	22.13 ^ab^	17.54 ^b^	4.9	0.001
Pleasantness of odour	37.38 ^b^	57.79 ^a^	58.17 ^a^	4.3	0.001
Typical pork flavour	49.21 ^b^	68.75 ^a^	70.46 ^a^	3.6	0.0001
Abnormal flavour	33.25 ^a^	23.17 ^ab^	15.13 ^b^	4.8	0.001
Pleasantness of flavour	46.96	59.21	56.17	4.4	0.332
Acceptability of sample	45.5	56.92	56.00	4.5	0.059

^1^ LSM—least squares mean. ^2^ SEM—standard error of the mean. Values with different superscripts within one row are significantly different at ^a,b^—*p* < 0.05.

**Table 3:** Evaluation of sensory preferences of *longissimus lumborum* in relation to different masking strategies at the skatole level of 0.269 μg/g.

Attribute	Control (n = 24)	*Allium sativum* (n = 24)	*Origanum vulgare* (n = 24)		
	LSM ^1^	LSM ^1^	LSM ^1^	SEM ^2^	*p*-Value
Typical pork odour	50.18 ^b^	71.04 ^a^	71.68 ^a^	3.3	0.0001
Abnormal odour	46.48 ^a^	31.00 ^b^	33.12 ^ab^	4.9	0.0003
Pleasantness of odour	36.68 ^b^	55.57 ^a^	48.00 ^ab^	4.3	0.0001
Typical pork flavour	41.64 ^b^	53.43 ^a^	53.27 ^a^	3.6	0.0001
Abnormal flavour	32.89	32.75	28.11	4.8	0.475
Pleasantness of flavour	40.57	45.39	43.11	4.4	0.530
Acceptability of sample	41.75	44.39	43.61	4.5	0.116

^1^ LSM—least squares mean. ^2^ SEM—standard error of the mean. Values with different superscripts within one row are significantly different at ^a,b^—*p* < 0.05.

**Table 4:** Evaluation of sensory preferences of *longissimus lumborum* in relation to different masking strategies at the skatole level of 0.463 μg/g.

Attribute	Control (n = 24)	*Allium sativum* (n = 24)	*Origanum vulgare* (n = 24)		
	LSM ^1^	LSM ^1^	LSM ^1^	SEM ^2^	*p*-Value
Typical pork odour	57.28	64.04	62.05	3.3	0.373
Abnormal odour	52.29	39.93	38.61	4.9	0.964
Pleasantness of odour	38.89	49.43	47.61	4.3	0.591
Typical pork flavour	52.89 ^b^	64.86 ^a^	61.86 ^ab^	3.6	0.021
Abnormal flavour	42.07 ^ab^	47.82 ^a^	28.11 ^b^	4.8	0.025
Pleasantness of flavour	41.82	42.54	51.50	4.4	0.115
Acceptability of sample	40.68	40.18	51.75	4.5	0.456

^1^ LSM—least squares mean. ^2^ SEM—standard error of the mean. Values with different superscripts within one row are significantly different at ^a,b^—*p* < 0.05.

**Table 5:** Evaluation of sensory preferences in relation to different masking strategies for neck chops.

Attribute	Control (n = 24)	*Allium sativum* (n = 24)	*Origanum vulgare* (n = 24)		
	LSM ^1^	LSM ^1^	LSM ^1^	SEM ^2^	*p*-Value
Typical pork odour	54.15 ^b^	63.70 ^ab^	67.85 ^a^	3.9	0.014
Abnormal odour	46.30	34.70	31.75	5.5	0.706
Pleasantness of odour	40.90 ^b^	55.10 ^a^	53.45 ^ab^	4.6	0.029
Typical pork flavour	50.10 ^b^	63.25 ^a^	62.25 ^ab^	4.5	0.032
Abnormal flavour	35.90	40.10	28.45	5.8	0.402
Pleasantness of flavour	48.10	47.60	49.01	5.1	0.836
Acceptability of sample	46.50	45.20	49.45	5.1	0.559

^1^ LSM—least squares mean. ^2^ SEM—standard error of the mean. Values with different superscripts within one row are significantly different at ^a,b^—*p* < 0.05.

**Table 6:** Evaluation of sensory preferences in relation to different masking strategies in *semimembranosus*.

Attribute	Control (n = 24)	*Allium sativum* (n = 24)	*Origanum vulgare* (n = 24)		
	LSM ^1^	LSM ^1^	LSM ^1^	SEM ^2^	*p*-Value
Typical pork odour	53.75 ^b^	75.60 ^ab^	78.05 ^a^	3.9	0.032
Abnormal odour	41.10 ^a^	16.50 ^b^	15.50 ^b^	5.5	0.002
Pleasantness of odour	46.90 ^b^	68.50 ^a^	62.65 ^a^	4.6	0.021
Typical pork flavour	50.70 ^b^	63.70 ^a^	67.40 ^a^	4.5	0.032
Abnormal flavour	30.75	29.00	17.14	5.8	0.366
Pleasantness of flavour	48.70	56.25	57.85	5.1	0.232
Acceptability of sample	50.60	55.70	60.75	5.1	0.073

^1^ LSM—least squares mean. ^2^ SEM—standard error of the mean. Values with different superscripts within one row are significantly different at ^a,b^—*p* < 0.05.

**Table 7:** Evaluation of sensory preferences of *Longissimus lumborum* in relation to different masking strategies.

Attribute	Control (n = 24)	*Allium sativum* (n = 24)	*Origanum vulgare* (n = 24)		
	LSM ^1^	LSM ^1^	LSM ^1^	SEM ^2^	*p*-Value
Typical pork odour	43.20 ^b^	67.00 ^a^	69.15 ^a^	3.9	0.0003
Abnormal odour	34.85	28.75	19.50	5.5	0.105
Pleasantness of odour	40.30 ^b^	58.65 ^a^	56.05 ^a^	4.6	0.006
Typical pork flavour	46.40 ^b^	58.95 ^a^	65.70 ^a^	4.5	0.013
Abnormal flavour	36.55 ^a^	27.45 ^ab^	20.30 ^b^	5.8	0.025
Pleasantness of flavour	42.55	53.80	52.50	5.1	0.443
Acceptability of sample	43.63	52.40	52.35	5.1	0.568

^1^ LSM—least squares mean. ^2^ SEM—standard error of the mean. Values with different superscripts within one row are significantly different at ^a,b^—*p* < 0.05.

**Table 8:** Evaluation of sensory preferences in relation to different masking strategies for subcutaneous fat.

Attribute	Control (n = 24)	*Allium sativum* (n = 24)	*Origanum vulgare* (n = 24)		
	LSM ^1^	LSM ^1^	LSM ^1^	SEM ^2^	*p*-Value
Typical pork odour	59.80	64.80	64.15	3.9	0.481
Abnormal odour	60.02	46.35	54.64	5.5	0.076
Pleasantness of odour	22.55	34.10	31.50	4.6	0.147
Typical pork flavour	44.20 ^b^	62.20 ^a^	50.40 ^a^	4.5	0.016
Abnormal flavour	41.65	44.05	39.15	5.8	0.085
Pleasantness of flavour	32.35	36.50	40.40	5.1	0.229
Acceptability of sample	29.25	33.40	38.15	5.1	0.088

^1^ LSM—least squares mean. ^2^ SEM—standard error of the mean. Values with different superscripts within one row are significantly different at ^a,b^—*p* < 0.05.

## Data Availability

None of the data were deposited in an official repository. The data that support the study findings are available to reviewers.
